# Colorectal cancer organoids: a developmental trajectory from Matrigel embedding to AI-empowered precision medicine

**DOI:** 10.3389/fonc.2026.1901112

**Published:** 2026-09-25

**Authors:** Muxin Tang, Lin Shaoyi, Qian Liang, Di Long

**Affiliations:** Wuming Hospital Affiliated to Guangxi Medical University, Nanning, China

**Keywords:** colorectal cancer, intelligent platforms, organoids, precision medicine, technological evolution

## Abstract

The high heterogeneity and complex microenvironment of colorectal cancer (CRC) represent critical bottlenecks contributing to treatment resistance and clinical translation failure, and the iterative development of organoid technology provides a key driving force for precision oncology. This narrative review systematically outlines the four-stage developmental trajectory of CRC organoid technology—while recognizing that these stages are not strictly sequential replacements but rather long-term coexisting and complementary approaches. The first-generation Matrigel embedding method established the foundation for three-dimensional culture but lacked adequate microenvironment simulation. The second generation achieved preliminary tumor–stromal interactions through optimized culture media and co-culture systems. The third generation integrated engineering technologies such as microfluidic chips and bioprinting, overcoming bottlenecks in high-throughput standardization and dynamic regulation in dedicated platforms. The fourth generation leverages artificial intelligence (AI), achieving substantial advances in intelligent phenotypic analysis, drug-response prediction, and initial exploration of digital twin systems. By comparing differences in heterogeneity retention, throughput, clinical potential, and cost-effectiveness among the four generations, this review emphasizes their core clinical value in individualized medication guidance and novel target discovery. Despite persistent challenges—including insufficient microenvironment simulation, poor AI explainability, and lack of standardization—current evidence suggests that interdisciplinary integration and intelligent platform development can transform organoid technology into a key hub bridging basic research and clinical practice. We therefore recommend accelerating the establishment of standardized organoid biobanks, embedding AI-driven predictive models into clinical workflows, and formulating regulatory frameworks to facilitate the translation of organoid-based precision medicine for colorectal cancer.

## 1 Introduction

Colorectal cancer, a high-incidence malignancy, is clinically challenging because of high heterogeneity, chemotherapy resistance, and complex metastatic mechanisms (1, 2). Conventional cell lines and patient-derived xenograft (PDX) models do not fully recapitulate the tumor microenvironment (TME) or patient-specific biological features, contributing to substantial translational discrepancies between preclinical findings and patient treatment responses (3, 4). Examples include resistance to targeted therapies mediated by KRAS mutations, epigenetic plasticity associated with oxaliplatin resistance, and relapse driven by residual cancer cells activating the MET-STAT3 pathway via LGR5+ subpopulations, highlighting the need for high-fidelity *in-vitro* models (5–7). In this context, organoid technology has emerged; it uses three-dimensional culture to retain histopathological features, genetic stability, and treatment responsiveness of the original tumor, supporting high-throughput drug screening and individualized treatment planning for precision medicine (8–11).

CRC organoid technology has progressed from traditional two-dimensional models to three-dimensional, self-organizing structures, overcoming limitations in the long-term culture of normal intestinal epithelial cells and improving the limited physiological relevance of animal models (12). Co-culture systems (e.g., with cancer-associated fibroblasts [CAFs], immune cells) can clarify how tumor–stroma interactions influence treatment response (13–15). Integration with engineering technologies such as microfluidic chips enables high-throughput drug screening (16). Incorporating single-cell technologies increases analytical resolution, deepening understanding of tumor heterogeneity and the mechanisms underlying treatment response (17). Collectively, these innovations advance individualized tumor therapy, support clinical efficacy prediction, and provide a platform for precision medicine, shifting basic research, drug development, and translational applications from static to dynamic models and from single-omics to integrated multi-omics approaches (18–20).

This narrative review aimed to systematically summarize and compare CRC organoid construction methods that have emerged over time, while recognizing that these approaches are not strictly sequential but often coexist in complementary ways. For instance, Matrigel-based culture remains the most widely used platform and serves as the foundation for many refinements described here. We evaluate their effectiveness in applications, describe current challenges, and forecast technological trends.

## 2 Generations of CRC organoid culture systems

A timeline of colorectal cancer organoid technology evolution and key milestones is shown in **Figure 1**.

**Figure 1:**
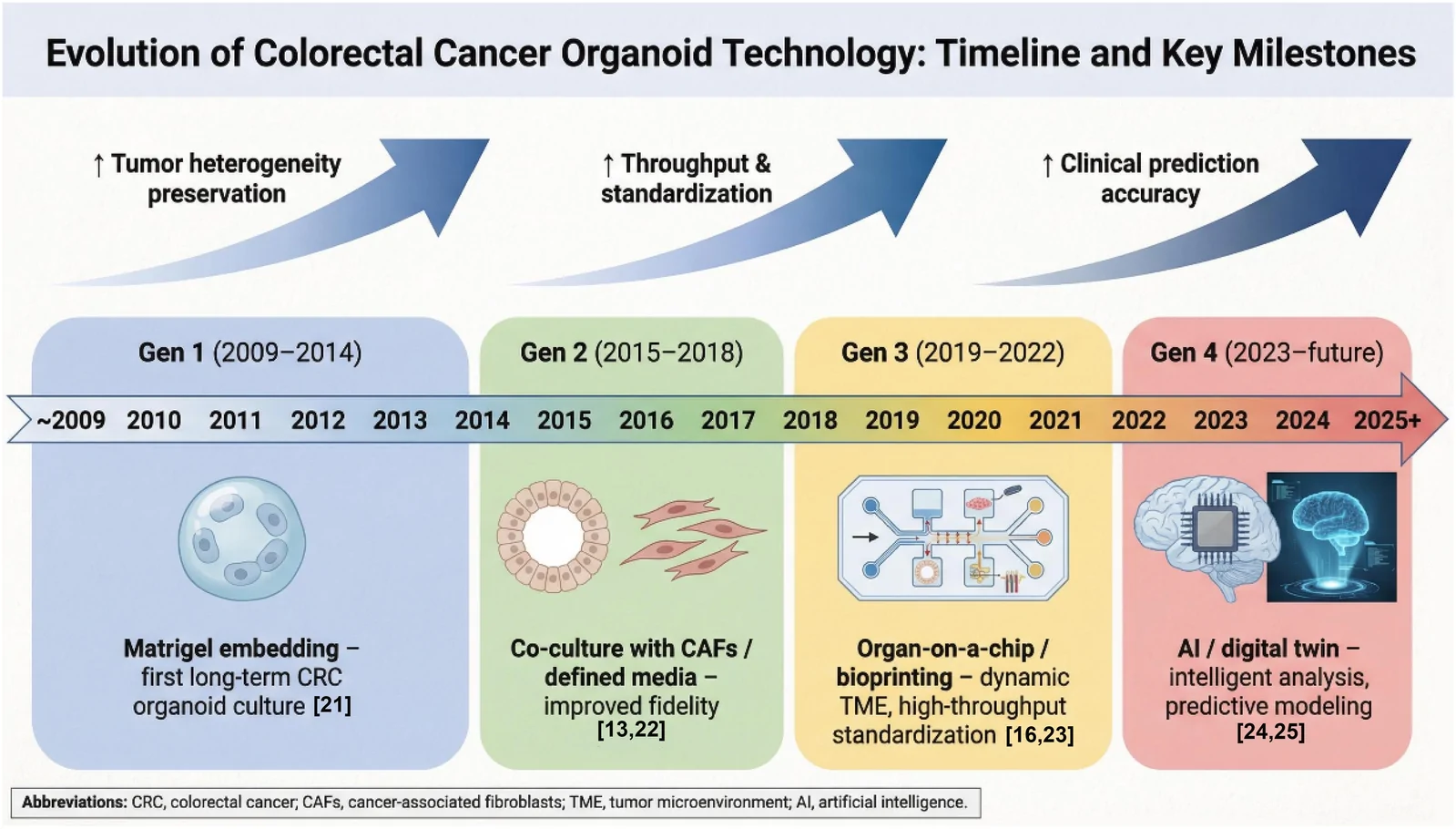
Timeline of colorectal cancer organoid technology evolution and key milestones. Four generations (Gen 1–4) are shown from ~2009 to the future. Gen 1: Matrigel embedding enables long-term culture of CRC organoids (21). Gen 2: Co-culture with CAFs and chemically defined media improves molecular fidelity (13, 22). Gen 3: Organ-on-a-chip and bioprinting introduce dynamic microenvironment control and high-throughput standardization (16, 23). Gen 4: AI and digital twin technologies enable intelligent phenotypic analysis and predictive modeling (24, 25). The upward arrows indicate progressive improvements in tumor heterogeneity preservation, throughput/standardization, and clinical prediction accuracy. CRC, colorectal cancer; CAFs, cancer-associated fibroblasts; AI, artificial intelligence.

### 2.1 First generation: classical Matrigel embedding system

#### 2.1.1 Principle and operational workflow

Matrigel-based organoid culture remains the most common method in CRC research and forms the basis for patient-derived biobanks. Although it is described here as a “first-generation” approach, this designation refers only to the chronological order of technological reports. The classical Matrigel embedding method uses Matrigel as the core scaffold, leveraging its extracellular matrix components, such as laminin and collagen, to mimic the *in vivo* basement membrane environment, providing adhesion cues and mechanical support, promoting tumor-cell aggregation into spheres and subsequent organoid formation. After enzymatic dissociation of CRC tissue into a single-cell suspension, cells are mixed with liquid Matrigel, seeded into culture plates (recommended density: 2000 cells/well), and gelled at 37 °C before addition of growth factor-supplemented medium. Stable structures form and maintain proliferative capacity within 25 days (21). For passaging, dispase digestion can preserve higher peptide yield and reduce matrix residue (26). This method represents the initial exploratory stage, with evaluation criteria including cell viability, culture success rate, and biological fidelity. The establishment of the first-generation Matrigel embedding system was a milestone, subsequently enabling the construction of patient-derived CRC organoid biobanks.

#### 2.1.2 High retention of tumor cell heterogeneity

CRC organoids generated using the traditional Matrigel embedding method retain the molecular and morphological heterogeneity of the original tumor (27, 28). Histological analysis reveals high similarity in glandular architecture, cell polarity, and nuclear-to-cytoplasmic ratio across organoids, patient primary tumors, and PDX (29). Genomic validation indicates that organoids stably preserve key driver gene mutations (e.g., *APC*, *KRAS*, *TP53*) and copy number variations present in the primary tumor (30). Transcriptomic analysis further confirms that tumor-derived organoids recapitulate the gene expression characteristics of *in vivo* cancer cells, including subtype-specific markers (e.g., genes associated with Consensus Molecular Subtype [CMS] classification) (31).

#### 2.1.3 Limitations

1. Batch-to-Batch Variability: Matrigel is an animal-derived extract with undefined components and substantial batch-to-batch variation, affecting experimental reproducibility (32). Proteomic analyses have identified 312 contaminating proteins (e.g., laminin subunits and collagens) in Matrigel; these contaminants can interfere with the accuracy of organoid proteomic data (26).
2. Uncontrollable Mechanical Properties: The stiffness and dynamic properties of Matrigel in its native form are difficult to tune, limiting its capacity to recapitulate tumor-specific mechanical microenvironments (33, 34).
3. Inadequate Microenvironment Simulation: It primarily supports epithelial cell culture and lacks key TME components (e.g., CAFs, immune cells, vascular endothelial cells), resulting in limited representation of cell-cell interactions (13). Furthermore, this model fails to reflect *in vivo* microenvironmental features such as hypoxia gradients and dynamic extracellular matrix remodeling, potentially leading to an overestimation of drug sensitivity (35). These limitations constrain clinical translation and have driven research to improve traditional methods.

### 2.2 Second generation: refined culture systems

Refinements to the classical Matrigel embedding system modify both the culture medium and matrix composition. Medium optimization mainly targets growth factor-free, chemically defined formulations. Research has evaluated synthetic hydrogels (e.g., gelatin-based or polyethylene glycol-based materials) and alternative natural matrices, and introduced co-culture techniques to improve the TME.

#### 2.2.1 Culture medium and matrix component refinements

A chemically defined medium that eliminates the need for exogenous Wnt, EGF, or BMP inhibitors—containing only five supplements (FGF10, A83-01, SB202190, gastrin, and nicotinamide)—has been developed. It enables long-term maintenance of organoid histopathological features, genetic stability, and treatment responsiveness without activating the Wnt/EGF pathway or inhibiting BMP signaling. This markedly lowers costs and improves reproducibility (22).

Although Matrigel remains the predominant matrix, a growing body of work is exploring natural matrices as alternatives. Patient-specific decellularized ECM can more closely recapitulate the metastatic TME, inducing greater tumor cell dissemination and migration in organoids. This matrix activates the TNF-α signaling pathway and AP-1 complex target genes (e.g., *MMP7*) and drives epithelial-mesenchymal transition (EMT) phenotypes (36). Laminin-enhanced decellularized small intestinal submucosa matrix (dSISML) supports CRC organoid growth and drug responsiveness and can be combined with engineering technologies for size-controlled, high-throughput production (16).

Significant research also focuses on synthetic matrices as alternatives to Matrigel. Similarly, synthetic matrices are under active investigation but have not yet achieved universal adoption. Enzymatically crosslinked synthetic hydrogels allow precise control of stiffness and adhesiveness in experimental settings, helping to mimic *in vivo* mechanical forces. PEG-based hydrogels permit precise stiffness modulation (~300 Pa–2 kPa): soft matrices (~300 Pa) markedly promote CRC colony formation, whereas stiff matrices (~2 kPa) strongly inhibit growth, and functional adhesive peptides (e.g., RGDS, DGEA) can be incorporated to regulate EGFR signaling (37). Gelatin-based hydrogels, although offering a narrower mechanical tuning range, naturally contain RGD motifs that provide inherent cell adhesion sites and are comparable to Matrigel in maintaining organoid viability, drug sensitivity, *in vivo* tumorigenicity, and supporting hypoxic metabolic adaptation (38). Both synthetic and semi-synthetic alternatives provide superior batch-to-batch consistency over undefined Matrigel.

#### 2.2.2 Microenvironment optimization

Co-culture integrates key cellular components to model tumor–stroma crosstalk, better recapitulating the TME and improving organoid fidelity. Co-culturing of CRC epithelial cells with stromal fibroblasts preserves tumor molecular features, supports proliferation, and improves cellular heterogeneity and morphological fidelity. These effects are mediated by cell–cell communication and ECM remodeling pathways, bringing the system closer to the *in vivo* microenvironment (39). CAFs have been found to promote cancer stemness and proliferation via paracrine signaling (e.g., hepatocyte growth factor-MET pathway), and blocking this interaction can inhibit oncogenic signaling (14). Single-cell transcriptomics shows that CAF co-culture improves transcriptomic fidelity and drives subtype-specific gene expression (e.g., CMS4 subtype features linked to gefitinib resistance) (13). Co-culturing organoids with peripheral blood lymphocytes, tumor-infiltrating lymphocytes, or macrophages both detects CRC-associated tumor factors (CEA, β-catenin, P53) and enables evaluation of immunotherapies (e.g., PD-1 inhibitors, CAR-T therapy) (40). Triple co-culture systems (PDOs, CAFs, myeloid cells) can generate a tumor-associated macrophage (TAM)-like phenotype, with CAFs enhancing chemotherapy-induced phagocytic activity (15).

These refined systems shift the focus from tumor cells alone to microenvironmental reconstruction, enabling partial TME simulation and significantly improving organoid viability and tissue fidelity. In parallel, optimization of culture media and the development of alternative scaffolds reduce culture costs and accelerate standardization efforts. However, standardization still requires further strengthening. Construction of complex microenvironments remains incomplete, and the controllability of the TME is limited.

### 2.3 Third generation: engineering and high-throughput platform construction

Third-generation CRC organoid construction technology features high-throughput, automated platforms. The integration of engineering approaches enables precise simulation and regulation of the TME, significantly improving construction efficiency, standardization, and physiological relevance. This provides technical support for large-scale drug screening and translational research.

#### 2.3.1 Organoid-on-a-chip

Organoid-on-a-chip technology primarily enables dynamic simulation and precise control of fluid dynamics; most platforms remain manually operated and are not intrinsically high-throughput (41, 42). However, specialized microwell chip designs have been developed to enable large-scale organoid production (43). High-throughput microwell chips (e.g., devices containing 30,000 microwells) support simultaneous large-scale generation of over 800 single cell-derived organoids, but these represent a minority of current systems and are often custom-built. In such platforms, robotic liquid handling and automated imaging are integrated to achieve true high-throughput workflows, substantially reducing manual intervention (44, 45).

Another important advance has been the construction of organoid-on-chip platforms that incorporate perfusable vascular networks. It is necessary to distinguish between endothelialized models, in which endothelial cells are incorporated without forming functional microvessels, and truly vascularized models, which contain perfusable, multicellular vessel-like structures. Furthermore, the spatial relationship between vessels and tumor cells critically influences data interpretation: vessels may be located (i) within the tumor spheroid/organoid interior, (ii) *de novo* within the organoid epithelium, or (iii) in the peri-spheroid stromal space. To date, most CRC organoid platforms—including the transparent microfluidic microtumor model (46) and the IFlowPlate system (47)—primarily generate perfusable vascular networks in the surrounding stromal compartment rather than within the tumor spheroid itself. There remains limited evidence that tumor cell–derived spheroids become internally vascularized. Consequently, results from such models should be interpreted as reflecting tumor–stromal vascular interactions rather than true intratumoral perfusion.

#### 2.3.2 Integration of organoid-on-a-chip with other technologies

Co-culture approaches can integrate CRC cells with stromal cells and microbiota to recapitulate a physiologically relevant TME, enabling visualization of key biological behaviors, including tumor cell proliferation, EMT, migration, and immune evasion (48, 49). For example, an organoid-on-chip co-culture system revealed that CAFs promote CRC cell proliferation and invasion through secreted exosomes, thereby reducing sensitivity to gefitinib (50). In a 3D environment, the cytotoxic effects of NK cell-derived exosomes have been observed and evaluated, with a particular advantage in targeting hypoxic tumor core regions (51).

Bioprinting enables precise spatial deposition of cells and biomaterials (e.g., viscoelastic hydrogels, composite bioinks), reconstructing physiologically relevant tumor tissue architectures *in vitro* (52, 53). Integrating organoid-on-a-chip with bioprinting further improves control of the microenvironment. Multivariate platforms combining both technologies (e.g., a 36-microenvironment model) can test 12 conditions, in parallel, capturing the complexity of TME mass transport and enabling efficient drug screening (54). This integration also allows the recapitulation of CRC progression and metastatic characteristics, strengthening vascular network integration and multicellular crosstalk within the models (55, 56).

Integrating microfluidic chips with hydrogel scaffolds to build a “mini-colon” prolongs tissue lifespan under asymmetric growth factor stimulation, and single-cell RNA sequencing confirms mature colonocyte features and spatial organization resembling *in vivo* conditions (23). A mini-colon platform based on microfabrication and optogenetics enables spatiotemporally controlled tumor induction and real-time analysis at single-cell resolution (57). These models recapitulate crypt-like structures (depth up to 65 µm) and mucus dynamics (58) and reproduce drug response characteristics; for example, the half-maximal inhibitory concentration of 5-fluorouracil for HCT116 tumor spheroids (540 ± 30 µm) is an order of magnitude higher than in 2D culture (42 ± 5 µm), more closely reflecting clinical drug resistance (59).

At this stage, organoid construction incorporates engineering approaches, shifting from manual handling to automated, high-throughput platforms. These systems allow dynamic control of the organoid microenvironment, more closely approximating *in vivo* physiological and pathological states. This shift enhances utility for drug screening and mechanistic studies and provides the data foundation for fourth-generation intelligent CRC organoids.

### 2.4 Fourth generation: intelligent engineering construction

The core of fourth-generation CRC organoid technology lies in the deep integration of artificial intelligence (AI), which substantially increases the precision of organoid analysis, enables intelligent organoid culture, improves drug screening prediction capabilities, and supports exploration of digital twin system construction, providing a powerful computational support platform for precision medicine (24).

#### 2.4.1 Analyzing organoid phenotypes

AI integrates high-throughput imaging with multi-omics data to support automated recognition, analysis, and deep quantification of CRC organoid morphology and function. Analyses of over five million images of organoids treated with small molecules revealed associations between morphological features and key signaling pathways: organoid size correlates with IGF1R signaling, and cystic structural features are significantly associated with LGR5+ stem cell status. This phenotypic quantification enables researchers to track the dynamic regulatory effects of drugs (e.g., MEK or mTOR inhibitors) on organoid morphology (60). A deeper analysis of 31,360 brightfield images identified two core CRC organoid morphological subtypes, cystic and solid, and object detection neural networks enabled precise single-organoid classification. The cystic subtype correlates with higher cell viability and expression of the intestinal stem cell marker LGR5, potentially serving as a marker for tumor recurrence. Deep generative models, trained on over 17,000 images, predicted apoptosis intensity, with a correlation as high as 0.91 between predicted and actual values, validating their feasibility and accuracy in assessing organoid function (61). Concurrently, deep learning-based convolutional neural network models (e.g., EfficientNet and U-Net) can predict microsatellite instability status from pathological images, a technology potentially extendable to organoid models (62).

#### 2.4.2 Dynamically regulating and optimizing culture parameters

AI is being explored to optimize CRC organoid culture, enabling intelligent, dynamic control of key culture parameters. Although no literature currently reports a direct application of AI to regulate CRC organoid culture parameters (63, 64), recent technological advances provide a foundation for future work. For instance, micro-engineered cell-culture devices and scalable automated suspension culture methods, coupled with high-content image-based phenotypic analysis, enable real-time monitoring and analysis of thousands of gastrointestinal organoid states, generating the data needed for feedback control systems (65). One study integrated ultra-thin film sensors with machine automation in an organoid culture system, creating an early adaptive culture platform. By sensing mechanical changes in the culture environment (e.g., indirect indicators of pH or metabolite accumulation), AI determines the optimal timing for medium exchange; machine automation, then executes the exchange, shifting from “static, fixed-interval” to “dynamic, on-demand” medium changes (66). In the future, AI is expected to use real-time, multimodal data (e.g., organoid morphology, growth rate) to dynamically adjust parameters such as nutrient/growth factor concentrations or physical stimuli, minimizing batch-to-batch variation and maximizing organoid states suited to specific research objectives (e.g., drug response testing).

#### 2.4.3 Drug screening and response prediction

AI-driven drug screening and response prediction algorithms are transforming personalized treatment decisions. Using computational prediction methods, 34 potential antitumor drugs were screened in CRC organoids, and transcriptomic analysis identified five drug-response signatures, including differentiation induction and growth inhibition. The efficacy of nintedanib, trametinib, and bortezomib was ultimately validated in organoid-based xenograft models (67). A deep learning-based model, STAR-CRC, integrating pathologic markers (e.g., pT4 stage, perineural invasion, and lymphovascular invasion), was used to construct the IRIS-CRC risk stratification system. In a cohort of 2,992 stage II patients, it performed exceptionally well, reclassifying 27.1% of high-risk patients into a low-risk group compared with traditional guidelines, accurately identifying 6.5% of patients with an extremely poor prognosis, and maintaining high generalizability across both chemotherapy-treated and untreated populations (68). A multimodal deep learning framework, FLARE, further integrates pathologic, radiologic, and clinical text data and accurately predicts survival prognosis in a cohort of 1,679 patients, significantly outperforming traditional models (69). These studies preliminarily demonstrate the effectiveness of combining AI with clinical and omics data to predict treatment outcomes and guide personalized therapy in CRC patients, providing a technical foundation for the subsequent construction of organoid-AI synergistic platforms for drug screening and response prediction.

#### 2.4.4 Construction of digital twin organoid systems

The rationale for constructing digital twin organoid systems is to build a virtual, dynamic replica of a physical organoid that enables real-time simulation, prediction, and optimization. At its core, this approach uses AI to integrate multisource data (25). Although direct digital twin model construction for CRC organoids remains in an early stage (e.g., projects such as ISPOT-K are exploring this), the underlying logic can be inferred from related technological trends. First, the system needs high-precision, high-throughput data acquisition, such as automated real-time analysis of brightfield images from thousands of organoids or whole-slide digital pathology scanning (64, 70, 71). Second, using the acquired multidimensional data (morphology, growth, and molecular data such as single-cell omics), deep learning models (e.g., CNN, U-Net, EfficientNet, FLARE) are applied for feature extraction and modeling (61, 68, 72). It must be emphasized that such digital twin systems remain at an early conceptual stage (25). The ultimate vision is a dynamic computational model that reflects the physical organoid state in real-time and predicts drug responses, creating an intelligent organoid platform with a “data-model-interaction” closed loop (24, 73). However, substantial obstacles—including multi-source data heterogeneity, lack of validated model-organoid correspondence standards, and immense computational demands (64)—must be overcome before clinical translation is feasible. Current efforts are limited to proof-of-principle studies and small-scale pilot projects (73); a fully functional digital twin for CRC organoids has not yet been realized.

The core modules of the fourth-generation AI-empowered colorectal cancer organoid platform are shown in **Figure 2**. “Perception” refers to real-time data acquisition (imaging, biosensors) capturing organoid morphology and microenvironmental parameters; “Regulation” denotes AI-driven feedback control that dynamically adjusts culture conditions (e.g., medium refresh timing, growth factor concentration). The central AI core integrates these streams for phenotypic analysis, drug response prediction, and digital modeling.

**Figure 2:**
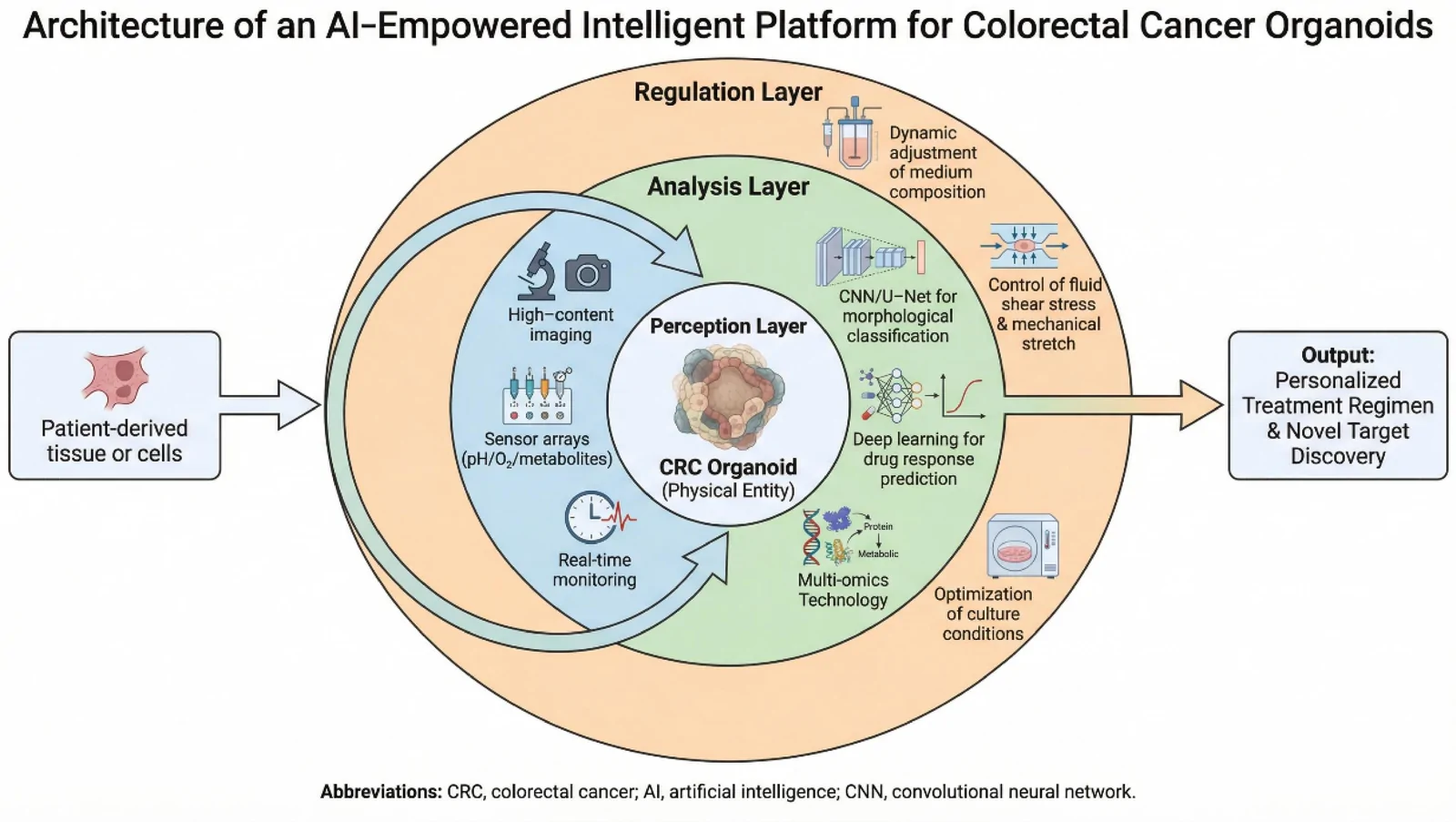
Core modules of the fourth-generation AI-empowered colorectal cancer organoid platform. The central intelligent platform integrates four interconnected modules: phenotypic analysis (CNN-based image analysis, morphological subtyping, apoptosis prediction), digital twin construction (multisource data integration, real-time virtual replica), drug screening and response prediction (deep learning, risk stratification), and dynamic culture optimization (real-time sensors, adaptive feedback). A circular arrow denotes the “Data → Model → Experiment → Optimization” cycle. An automated culture system (microfluidics, bioprinting) supports the platform. Outputs include personalized treatment regimens and novel target discovery. AI, artificial intelligence; CNN, convolutional neural network.

## 3 Comparison of application efficacy of different construction methods

A comparison of the core characteristics of four generations of colorectal cancer organoid construction technologies is shown in **Table 1**.

**Table 1:** Comparison of core characteristics of four generations of colorectal cancer organoid construction technologies.

Generation	Core construction method	Key representative technologies	Tumor microenvironment modeling capacity	Throughput & standardization level	Major limitations
1st	Matrigel embedding	Simple 3D culture in Matrigel droplets (21, 23)	Minimal (epithelium only)	Low throughput; high batch-to-batch variability (22, 28)	Animal-derived matrix; uncontrolled stiffness; lack of stromal/immune cells; poor reproducibility (13, 29–31)
2nd	Modified culture systems	Growth factor-reduced chemically defined medium (32); natural matrix and synthetic hydrogels (33, 34); co-culture techniques (14, 36)	Partial [introduction of CAFs and some immune cells via co-culture (15, 37)]	Medium throughput; improved but still largely manual (32, 76)	Incomplete TME reconstruction; insufficient standardization of co-culture ratios and matrix substitutes (13)
3rd	Engineering & high-throughput platforms	Organ-on-a-chip (16, 38–40); bioprinting (49, 51); mini-colon (54, 55); vascularized microtumors (43, 44)	Dynamic [fluid shear stress, mechanical stretch, vascularization (54–57)]	High throughput; automated; high standardization (16, 64)	High equipment cost; complex operation; need for full integration of vascular/immune components
4th	AI-empowered intelligent platform	Deep learning (CNN, U-Net) (59–61); digital twin (69, 73); multi-omics integration; intelligent feedback control (64, 65)	Predictive and dynamic [AI-inferred interactions, digital modeling (66–68)]	Ultra-high throughput; intelligent and automated (64, 65)	Poor AI explainability; requirement for large training datasets; ethical/regulatory gaps

Comparison of four generations of colorectal cancer organoid construction technologies. Key features include the core construction method, representative technologies, tumor microenvironment modeling capacity, throughput and level of standardization, and major limitations. Representative references are provided in brackets and correspond to the reference list in the main text. CRC, colorectal cancer; CAFs, cancer-associated fibroblasts; TME, tumor microenvironment; AI, artificial intelligence; CNN, convolutional neural network.

### 3.1 Intergenerational evolution of tumor heterogeneity retention capability

The first-generation Matrigel embedding method can already preserve histopathological grade, differentiation capacity, and histological features resembling those of the primary tumor (21, 23, 74). Nonetheless, its simplified microenvironment limits the simulation of more complex heterogeneity (4, 13). By introducing multicellular co-culture systems (e.g., integrating CAFs), second-generation technology significantly enhances fidelity to tumor molecular subtype characteristics (e.g., CMS4 subtype), more accurately reflecting inter- and intra-tumor heterogeneity, resistance mechanisms, and prognostic expression profiles (13). Third-generation engineered construction technologies (e.g., organoid-on-a-chip) maintain independent organoid growth while avoiding heterogeneity introduced by solid matrices, thereby ensuring molecular and functional stability during long-term culture and drug screening (16, 48). Fourth-generation AI-empowered intelligent construction uses machine learning to analyze large volumes of organoid image and omics data, enabling in-depth analysis of the dynamic evolution of tumor heterogeneity (e.g., clonal dynamics and evolutionary patterns) and epigenetic regulatory mechanisms, and offering new dimensions for understanding the essence of heterogeneity (24, 75).

### 3.2 Comparison of throughput and standardization levels

First-generation technology typically uses 96-well plate culture (e.g., 2000 cells/well), limiting throughput and standardization (21). Second-generation technology improves reproducibility through optimized culture media (e.g., growth factor-free media) and matrix materials (e.g., synthetic hydrogels), but standardization is still restricted by manual handling (22, 76). Third-generation technology marks a qualitative leap: combining microfluidic chips (e.g., laminin-enhanced dSISML platform), bioprinting, and related tools enables automated, high-throughput production of thousands of uniformly sized organoid microspheres in microcavity arrays, increasing construction throughput and processing efficiency, and improving the standardization and scalability of drug screening (16, 64). Fourth-generation technology further leverages AI algorithms (e.g., image-based high-content phenotypic analysis) to automate the processing and analysis of large organoid datasets, optimize culture conditions, and predict drug responses, enabling higher-level standardization and more intelligent data acquisition and analysis (64, 65).

### 3.3 Gradient differences in clinical application potential

First-generation technology is used mainly for basic research, showing that organoids can retain tumor features and support drug sensitivity testing. Its clinical application (e.g., personalized therapy selection) remains in preliminary exploration (3, 21, 24). By enabling more realistic TME simulation (e.g., co-culture systems) and optimized culture conditions, second-generation technology improves the model’s ability to predict clinical treatment responses (e.g., chemotherapy, targeted therapy resistance). This supports more reliable personalized treatment recommendations and reduces overtreatment, thereby increasing clinical translation potential (13, 77, 78). Third-generation technology enables high-throughput, standardized production, making it well-suited for large-scale drug screening and “phase 0” human trials. Constructed models (e.g., orthotopic patient-derived organoid xenografts, PDOX) can accurately simulate tumor heterogeneity and maintain consistent treatment responses *in vivo* and *in vitro*, providing quantitative evidence for individualized treatment decisions and establishing standards for drug sensitivity assessment, further enhancing clinical utility (16, 78, 79). Fourth-generation technology integrates AI analysis with predictive models, enabling detailed analysis of resistance mechanisms, prediction of combination therapy strategies (e.g., EGFR/KRAS dual inhibition, AURKA inhibition), and guidance for precise medication. It demonstrates the highest level of clinical application potential for dynamic treatment monitoring and individualized precision treatment planning (25, 66, 75).

### 3.4 Cost-effectiveness analysis

First-generation technology relies on costly, batch-variable Matrigel, which increases costs, and standardization challenges also limit its cost-effectiveness. Second-generation technology lowers media costs by developing chemically defined, growth-factor-free media (e.g., FGF10 and A83-01) and by exploring alternative matrices (e.g., synthetic hydrogels), thereby reducing reliance on biological matrices. By improving tumor heterogeneity retention and the accuracy of drug response prediction, it increases the model’s practical value, and achieves a favorable cost-effectiveness balance (22). Although third-generation technology requires a higher upfront investment (e.g., microfluidic chips and bioprinting equipment), its automated, high-throughput workflows reduce per-organoid costs and improve drug-screening efficiency, and standardized production minimizes experimental variability and repeat costs. From a long-term, large-scale application perspective, it offers higher cost-effectiveness (16, 64). Fourth-generation AI technology adds costs for algorithm development and computational resources, but it can enhance R&D efficiency and treatment success rates by optimizing experimental design (e.g., reducing trial and error), accurately predicting outcomes (e.g., drug responses and optimal treatment combinations), and extracting greater value from complex datasets. Thus, it holds potentially optimal cost-effectiveness in precision medicine applications, especially for complex cases and expensive targeted/immunotherapy regimens (75, 80). As a preliminary comparison, the estimated cost per organoid culture using traditional Matrigel-based methods is approximately $50–100 per well (including growth factors), whereas synthetic hydrogel matrices can reduce matrix costs by 60–80% (22, 38). Third-generation microfluidic platforms, despite higher initial equipment investment (~$10,000–30,000), can process thousands of organoids in parallel, reducing the per-data-point cost to roughly $5–10 in consumables. Although AI integration adds computational expenditure, it can significantly lower failure rates and minimize repeated experiments, making it economically advantageous for large-scale, high-stakes screening contexts. comparison of four generations of colorectal cancer organoid technologies across key application efficacy dimensions is shown in **Table 2**.

**Table 2:** Comparison of four generations of colorectal cancer organoid technologies across key application efficacy dimensions.

Generation	Representative technologies/features	Tumor heterogeneity preservation	Clinical application potential	Cost-effectiveness
1st	Matrigel-based 3D culture (21, 23)	Moderate (preserves basic histopathological features but lacks stromal/immune components) (24, 26)	Low (primarily for basic research) (3, 21, 24)	Low (expensive Matrigel and batch variability increase repeat costs) (21, 22)
2nd	Modified medium/matrix + co-culture (e.g., with CAFs) (13, 32)	High (improved fidelity of molecular subtypes, e.g., CMS4 subtype) (13, 36)	Moderate (can support personalized treatment recommendations) (13, 77, 78)	Moderate (reduced medium cost but complex co-culture operation) (32, 76)
3rd	Organ-on-a-chip, bioprinting, vascularized microtumors (16, 48)	High (dynamic microenvironment maintains molecular and functional stability) (16, 79)	High (suitable for large-scale drug screening, Phase 0 clinical trials) (16, 78, 79)	Fairly high (high initial equipment investment but reduced unit production cost) (16, 64)
4th	AI deep learning, digital twin, multi-omics integration (58, 75)	Extremely high (AI-deciphered clonal evolution, epigenetic dynamics) (58, 75)	Extremely high (dynamic treatment monitoring, digital twin prediction) (66, 69, 75)	Optimal (reduced trial-and-error, precise prediction; significant value for complex cases) (75, 80)

Comparison of four generations of colorectal cancer organoid technologies across key dimensions of application efficacy, including tumor heterogeneity preservation, clinical application potential, and cost-effectiveness. Representative technologies and references are provided for each generation. CRC, colorectal cancer; CAFs, cancer-associated fibroblasts; CMS4, consensus molecular subtype 4; AI, artificial intelligence.

To complement the qualitative intergenerational comparison, we systematically summarized the quantitative predictive performance reported across different organoid platforms. First-generation Matrigel-based organoid drug testing predicted response to irinotecan-based chemotherapy in metastatic colorectal cancer patients with a sensitivity of approximately 80%, specificity of 70–75%, and accuracy of around 83% (90). Second-generation co-culture platforms, such as those incorporating cancer-associated fibroblasts, improved concordance with clinical outcomes for targeted agents, with a reported positive predictive value of approximately 86% (13). Third-generation organ-on-chip systems demonstrated high-throughput consistency, with a batch-to-batch coefficient of variation below 15% and a correlation coefficient of 0.89 with *in vivo* efficacy (16); during the same period, a phase II trial (NCT03251612) using organoid-guided therapy reported a 2-month progression-free survival rate of 50% (91). Although fourth-generation AI-assisted screening has not yet yielded direct clinical predictive values, it has achieved a correlation coefficient of 0.91 between predicted and actual apoptosis intensity (61). Overall, directly comparable data across generations remain limited, but published results suggest a trend toward improved predictive accuracy with more sophisticated models, which nonetheless requires confirmation through prospective multicenter studies.

## 4 Clinical applications

Leveraging their ability to preserve the genetic and phenotypic characteristics, as well as the heterogeneity of the original tumor, CRC organoid models have promising prospects for clinical translation. Their core applications include supporting the discovery of novel diagnostic and therapeutic targets and formulating personalized treatment strategies (81, 82).

### 4.1 Discovery of novel diagnostic and therapeutic targets

Organoid models integrated with multi-omics analyses have become effective platforms for target discovery. Combined proteomics (SWATH-MS) and transcriptomics (RNA-seq) demonstrated that dependence on oxidative phosphorylation and enrichment of tRNA aminoacylation are associated with oxaliplatin resistance, whereas MYC activation and TRiC complex enrichment correlate with palbociclib hypersensitivity (83). Genomically, homologous recombination deficiency was found to predict oxaliplatin sensitivity and was validated both *in vitro* and *in vivo* as a potential biomarker for recurrence-free survival (84). In epigenetic regulation, chromatin accessibility analysis (ATAC-Seq) identified patient-specific targets associated with oxaliplatin resistance (e.g., *FGFR1*/*OXTR*); their inhibitors could reverse the resistant phenotype (6). Loss of Suv4-20h2-mediated H4K20me3 in right-sided CRC organoids was identified as a subtype-specific therapeutic target (85). Mechanistic studies showed that CK1δ/ϵ promotes Wnt/Notch signaling activation by phosphorylating and degrading the tumor suppressor AES. The inhibitor SR3029 suppressed organoid growth and metastasis (86). Furthermore, liquid biopsy combined with organoid drug sensitivity testing confirmed enhanced sensitivity to alpelisib in patients with *PIK3CA* mutations (87). The selective cytotoxic effect of BET inhibitors and the association of *CHFR* silencing with paclitaxel sensitivity provide new evidence for precision therapy (88).

### 4.2 Assisting in formulating individualized treatment plans

CRC organoid models are being actively evaluated as promising *in vitro* tools to support individualized treatment planning; however, their clinical effectiveness is still being established in prospective trials, and routine implementation is not yet standard. When applied in clinical settings, including postoperative adjuvant chemotherapy, palliative chemotherapy, neoadjuvant chemoradiotherapy, and targeted therapy, they can accurately predict patient responses to different drug regimens, thereby guiding personalized medication and reducing toxicity and resistance risks (89). Prospective clinical studies show that *in vitro* drug sensitivity testing using metastatic CRC (mCRC) patient-derived organoids (PDOs) achieves over 80% predictive accuracy for irinotecan-based chemotherapy regimens (90). A phase II clinical trial (NCT03251612) further validated the feasibility: when PDO-based drug sensitivity results were used to guide therapy, 50% (17/34) of patients achieved progression-free survival at 2 months, with a median progression-free survival of 67 days and a median overall survival of 189 days (91). In palliative care patients, PDO drug testing demonstrated 83% predictive accuracy for standard-of-care regimens (92). Notably, PDO models efficiently predict targeted therapy efficacy: *RAS* wild-type PDOs were sensitive to cetuximab (83.3% response rate), whereas *RAS* mutant PDOs were uniformly resistant (100% resistance). Adjusting treatment regimens based on drug sensitivity testing achieved disease control in chemotherapy-resistant patients (93). Additionally, a high-throughput screening platform based on PDOs successfully identified antitumor effects of drugs such as temozolomide, trametinib, and bortezomib (66). Using a half-maximal effective concentration gradient classification method enabled chemosensitivity/resistance to be determined within 1 week, with results highly consistent with patient clinical responses (94).

## 5 Current challenges and limitations

Despite major advances in CRC organoid technology, technical bottlenecks and controversies still constrain clinical translation and widespread application, primarily concerning TME simulation, AI integration, standardization, and regulatory frameworks.

### 5.1 Insufficient tumor microenvironment simulation

Although organoid technology has advanced in capturing key CRC epithelial elements, it still cannot fully replicate the dynamic, multicellular interactions of the *in vivo* TME, particularly immune crosstalk and vascular networks. Regarding immune microenvironment simulation, PDOs often do not recapitulate the primary tumor’s immune phenotype, leading to the absence of immune-related pathways (e.g., immune cell infiltration, cytokine signaling) (95). Co-culture systems (e.g., adding CAFs or myeloid cells) can partly reconstitute the microenvironment and support immune-related functions (e.g., macrophage polarization, phagocytic activity) (15, 36, 96), but reconstructing complex *in vivo* neuro-immune interactions, metabolic reprogramming, and the dynamic regulation of immunosuppressive cells (e.g., MDSCs, TAMs) remains highly challenging (97–101). Inadequate vascularization simulation leads to internal hypoxia and limited nutrient delivery, directly affecting organoid viability, growth, and the physiological relevance of drug response assessments (102, 103). Although organoid-on-a-chip platforms can initially construct functional vascular networks and enable immune cell perfusion, these methods are complex to implement and not yet standardized (44, 104–106). Collectively, these gaps suggest that coordinated integration of vascular and immune components remains a key direction for future technological breakthroughs (107, 108).

### 5.2 AI model explainability and clinical acceptance

AI exhibits substantial potential for organoid phenotypic analysis and drug prediction, but clinical translation is hampered by limited model explainability and acceptance. Although machine learning approaches can analyze organoid growth dynamics from morphological features, the biological mechanisms inferred by these models (e.g., correlations between proliferation rate and microenvironmental factors) often remain “black box” outputs, making the underlying biology difficult to interpret (109, 110). Here, “explainability” refers to the ability to understand how a model arrives at a specific decision or prediction in terms that are biologically meaningful (24, 109). For example, a convolutional neural network may classify an organoid as “drug-resistant” based on uninterpretable texture features, without revealing which signaling pathways or genetic alterations drive the resistance (60). Similarly, a drug response prediction model might output a sensitivity score without indicating whether it acts through apoptosis or cell cycle arrest, limiting its clinical credibility (61). On this basis, studies of nanohybrid-treated organoids validate efficacy, but insufficient AI-driven mechanistic analysis may impact trust in clinical decision-making (111). For drug sensitivity prediction, deep learning models may exhibit unstable performance across centers and limited adaptability to rare mutation types or specific subtypes (112, 113). Clinical adoption of AI-predicted results still requires validation in large-scale prospective studies to confirm reliability, particularly in treatment scenarios involving dynamic changes in the immune microenvironment (e.g., immune checkpoint inhibitor responses) (114). AI-driven dynamic monitoring systems (e.g., digital twin technology) have yet to resolve standardization challenges in multimodal data integration, which restricts clinical translation (115).

### 5.3 Barriers to establishing standardized systems

CRC organoid technology standardization faces several structural barriers. First, culture protocols are not harmonized: basal media formulations (e.g., need for Wnt/EGF pathway activation) and matrix materials (e.g., Matrigel, dSISML, synthetic hydrogels) vary substantially, directly affecting retention of organoid heterogeneity and reproducibility (16, 22, 96). Second, operational workflows differ across laboratories. From tissue procurement and seeding density (2000 cells/well) to co-culture ratios (e.g., CAF and immune cell ratios) and spatial configuration, consensus protocols are lacking (13, 21, 99). Third, quality assessment remains underdeveloped: organoid vascularization and immune cell infiltration efficiency lack quantitative benchmarks (105, 116), and multi-omics integration (e.g., single-cell transcriptomics, metabolomics) is constrained by inconsistent analysis pipelines (117, 118). Collectively, these issues reduce cross-study comparability and slow validation of organoids as clinical predictive tools. Although guidelines such as the “Human Intestinal Cancer Organoid” protocol represent an initial step, further refinement is required, particularly as AI-based approaches are incorporated, and broad adoption will take time (119).

### 5.4 Lack of ethical norms and regulatory frameworks

The clinical translation of organoid technology lacks systematic ethical and regulatory frameworks. Although organoids support individualized treatment decisions and “phase 0” clinical trials, genetic drift during long-term culture raises concerns about result reliability (26). Microenvironment engineering relies on biomaterials (e.g., decellularized matrices, synthetic scaffolds), yet clinical safety assessment lacks standardized quality control processes (120). Although multi-cell co-culture systems (e.g., triple models including CAFs and immune cells) enhance microenvironmental fidelity, ethical boundaries regarding the sourcing of allogeneic cells remain ambiguous (15). Furthermore, informed consent mechanisms for organoid biobanks need to clarify tissue ownership and the scope of data sharing (121, 122), and deeper integration of organoid technology with AI and engineering (e.g., bioprinting, organ-on-a-chip) introduces new ethical challenges.

## 6 Future perspectives and development trends

Continued CRC organoid construction technology innovation will prioritize greater physiological fidelity, improved predictive accuracy, and more efficient clinical translation. Key directions include deeper integration of multi-omics technologies, development of automated intelligent platforms, construction of individualized models, and refinement of ethical and regulatory systems, establishing organoids as a central hub linking basic research, drug development, and clinical practice.

### 6.1 Integration of multi-omics technologies with organoid models

CRC organoids faithfully recapitulate the genomic, transcriptomic, and phosphoproteomic profiles of the original tumor, maintaining histopathological characteristics and genetic stability of primary tumors during long-term culture, providing an ideal platform for multi-omics analyses (26, 27). Deep integration of multi-omics technologies (e.g., genomics, transcriptomics, proteomics, epigenetics) enables comprehensive assessment of tumor heterogeneity, microenvironmental interactions, and molecular mechanisms, thereby improving biological fidelity and clinical predictive value (83, 123, 124). For example, integrating drug sensitivity testing with proteomics (SWATH-MS) and transcriptomics (RNA-seq) reveals resistance mechanisms, including enrichment of tRNA aminoacylation and reliance on oxidative phosphorylation in platinum non-responders (83). Combining chromatin accessibility analysis (ATAC-Seq) with transcriptomics identified activation of *FGFR1*/*OXTR* pathways associated with oxaliplatin resistance, providing targets to overcome resistance (6). Spatial multi-omics technologies (e.g., SM-Omics) enable spatially resolved combined transcriptomic and proteomic analyses in tissues, potentially supporting organoid microenvironment simulation (125). Cross-omics interaction frameworks (e.g., multi-omics pre-trained network TMO-Net) can better represent incomplete multi-omics data and clarify how different omics features contribute to clinical outcomes (126). Advanced statistical methods (e.g., MDICC, moiraine R package) improve cancer subtype identification and survival prediction accuracy via similarity matrix fusion and network integration (127, 128). Future development requires more efficient multi-omics fusion algorithms and standardized integration workflows to accurately identify key pathways driving tumor progression and personalized therapeutic targets (129, 130).

### 6.2 Precision construction in personalized medicine

Precisely constructing organoids according to patient molecular subtyping is crucial for accelerating translation in personalized medicine. Integrating multi-omics data enables organoid models to capture CRC molecular subtype characteristics (e.g., three proteomic subtypes) and provides a biological rationale for optimizing treatment strategies: the S_I subtype is sensitive to alisertib, whereas C1-C4 TME subtypes are suited to chemotherapy, targeted therapy, and immunotherapy regimens (131, 132). In clinical applications, combining liquid biopsy with organoid drug sensitivity testing can screen patients likely to benefit from targeted therapies; for example, those with *PIK3CA* mutations show significantly enhanced sensitivity to alpelisib (92). To further enhance predictive power, AI models integrating organoid drug sensitivity data with patient genomic information can be developed to predict individualized treatment responses. Organoid-on-a-chip platforms can evaluate chemotherapy sensitivity or the anti-tumor effects of novel nanomaterials (e.g., M-MoS2 nanosheets), providing preclinical evidence to inform clinical medication (133, 134). Future efforts should focus on establishing large-scale, multi-center organoid biobanks that integrate clinical data and multi-omics features, developing organoid evolution models, formulating dynamic treatment strategies, and enabling “digital twin” technology to guide clinical decisions (135, 136).

### 6.3 Development of automated intelligent platforms

Automated intelligent culture systems are central to overcoming barriers in standardization and scalability. Engineering approaches such as bioprinting and microfluidic chips enable standardized organoid expansion and dynamic microenvironment control, supporting high-throughput applications, whereas machine learning algorithms can optimize culture conditions and improve morphological analysis efficiency (16, 31). Currently, closer integration of AI and engineering is pushing platforms toward intelligent operation: AI can monitor organoid morphology and gene expression dynamics in real time (e.g., analyzing temporal expression patterns using Bayesian frameworks), while microfluidic chips dynamically regulate culture parameters, improving physiological simulation fidelity (137, 138). Integrating these platforms with multi-omics technologies also expands the sensing dimension. For example, chemical tomography combined with graphene sensor arrays can monitor volatile organic compounds from organoids, enabling real-time analysis of cell-protein-genome multi-omic features to distinguish between normal and diseased states (139). Building on this, machine learning algorithms (e.g., FSD feature dimensionality reduction and the Attention MOI framework) can enhance multi-omics data performance for survival prediction and subtype identification (140). Future work involves integrating automated culture, high-throughput drug screening, real-time imaging, and multi-omics data analysis to construct an intelligent platform with integrated “culture-test-predict” functions, However, it is important to note that the digital twin paradigm, while inspiring, is still in its infancy (25, 123); bridging the gap between current organoid models and clinical decision-support tools will require extensive validation (141, 149), standardized data pipelines (119, 128), and resolution of ethical-regulatory challenges (73, 143). This integrated platform is more realistically a medium-to-long-term goal rather than an immediately deployable technology (24, 135).

### 6.4 Regulatory frameworks and technological ethics

The clinical translation of organoid technology requires robust regulatory frameworks and addressing ethical challenges to support compliant use. Organoid biobanks face reproducibility and scalability issues, requiring uniform quality control standards for culture, standardized phenotypic assessment protocols, and data sharing agreements (141, 142). Future efforts should bring together research institutions, ethics committees, and regulatory bodies to develop guiding principles that balance technological innovation with ethical safety. This includes strengthening patient data protection mechanisms, clarifying ownership of organoid-derived data, and ensuring explainability of AI-driven organoid analyses (e.g., morphology recognition and treatment prediction) to avoid “black-box” decision-making. Concretely, this means that when an AI system flags a morphological feature as indicative of LGR5+ stemness or predicts resistance to cetuximab, it should ideally highlight the contributing image regions or molecular signatures, rather than providing a numerical score alone (60). Without such transparency, clinicians cannot confidently integrate AI recommendations into treatment planning (114). Legal responsibility in diagnostic reports should also be defined should AI-assisted recommendations prove erroneous. Additionally, limited diversity in training data can lead to algorithmic bias, necessitating multi-center collaborations to ensure data representativeness (143, 144).

A summary of the current challenges and future solutions for colorectal cancer organoid technology is shown in **Figure 3**.

**Figure 3:**
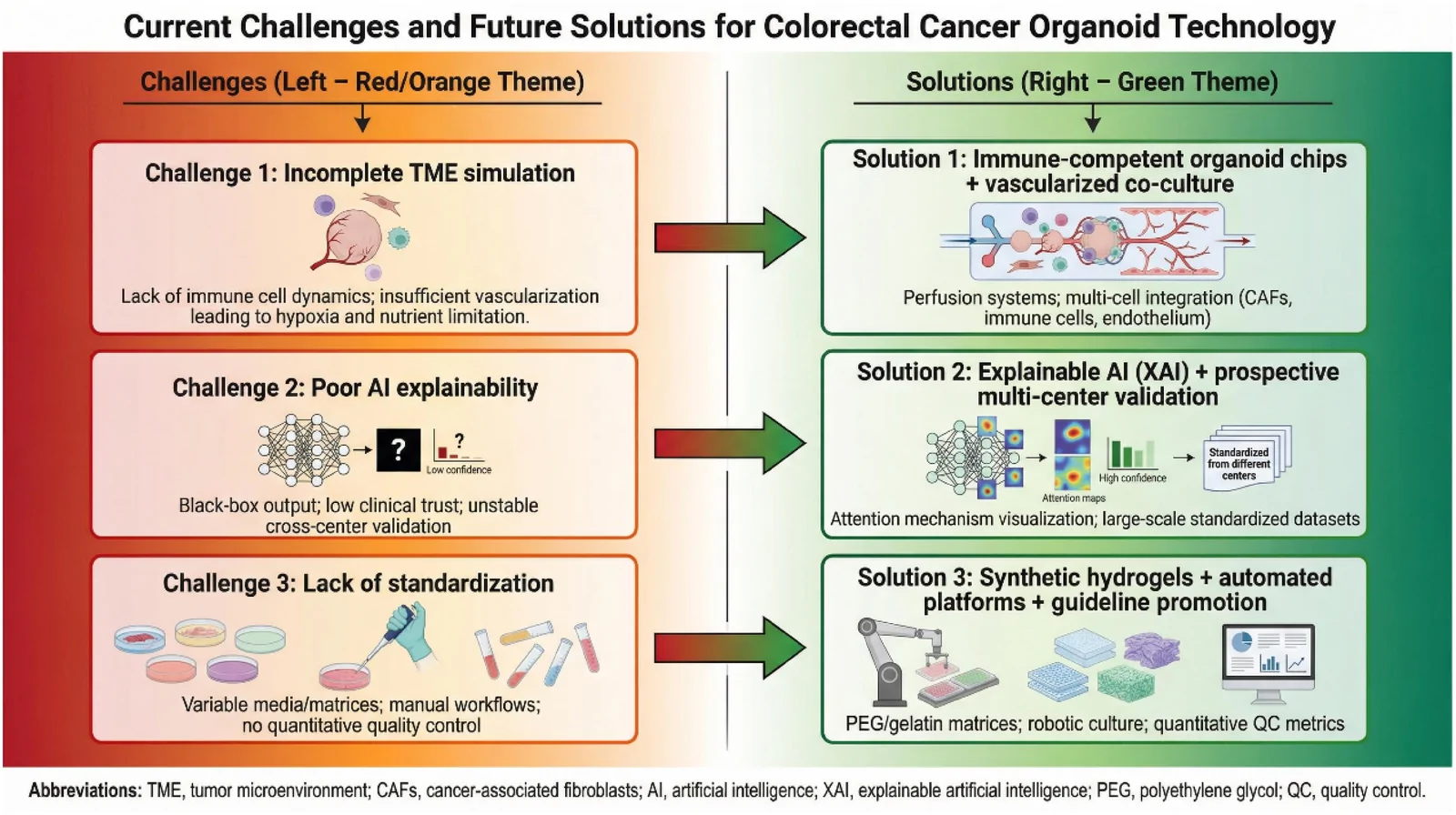
Current challenges and future solutions for colorectal cancer organoid technology. The left panel highlights three major challenges: incomplete tumor microenvironment (TME) simulation (limited immune dynamics and vascularization), poor AI explainability (black-box outputs, low clinical trust), and insufficient standardization (variable media/matrices, manual workflows). The right panel presents the corresponding solutions: immune-competent organoid chips with vascularized co-culture, explainable AI (XAI) with prospective multicenter validation, and synthetic hydrogels combined with automated platforms and guideline promotion. Horizontal arrows connect each challenge to its corresponding solution. TME, tumor microenvironment; AI, artificial intelligence; XAI, explainable artificial intelligence; PEG, polyethylene glycol; QC, quality control.

## 7 Conclusion

### 7.1 Core patterns of technological evolution

The four-generation evolution of CRC organoid technology has shifted from basic culture systems to precision, high-throughput, and intelligent development. As the field progressed from Matrigel embedding to AI integration, the core change has been stepwise strengthening of the three-dimensional attributes of organoid models: “fidelity, high-throughput standardization, and predictability.”

First-generation Matrigel embedding enabled three-dimensional primary culture of tumor tissue but was limited by reduced cellular heterogeneity and a simplified microenvironment (4). Second-generation technology improved TME simulation by developing growth factor-reduced media (e.g., FGF10, A83-01), matrix alternatives, and multicellular co-culture systems, improving organoid viability and shortening culture duration (13, 22, 35). Third-generation engineering delivered major advances: organoid-on-a-chip supported dynamic culture and produced uniform organoids, enabling high-throughput standardized screening platforms. This facilitated automated, scalable organoid production, reduced batch variability, and laid the foundation for large-scale drug screening (16, 64). Integration with bioprinting and co-culture enabled simulation of tumor invasion (79). Fourth-generation AI-empowered intelligent construction uses machine learning to optimize culture conditions, analyze organoid phenotypes, and predict drug responses, and, combined with multi-omics data (e.g., transcriptomics, chromatin accessibility), constructs digital models, driving the technology toward intelligence and precision (6, 78, 145).

### 7.2 Core value of clinical translation

The core clinical value of CRC organoid models lies in their ability to closely recapitulate the histologic features, molecular heterogeneity, genomic characteristics, and treatment responsiveness of the patient’s original tumor, making them valuable tools for individualized treatment planning, clinical efficacy prediction, and new drug development (3, 24, 66, 146). Clinical evidence demonstrates that organoid drug sensitivity testing can effectively guide clinical decisions: PDOs/patient-derived tumor organoids can predict, with high accuracy, sensitivity to chemotherapy (e.g., 5-fluorouracil, irinotecan), targeted therapy, and radiotherapy, with *in vitro* drug and radiation sensitivity correlating with patient outcomes, potentially guiding a reduction in drug toxicity and resistance risks (89, 147–149). A phase II clinical trial (NCT03251612) further confirmed translational feasibility and value, showing that organoid-based *in vitro* sensitivity results guided precision treatment and achieved a 2-month progression-free survival rate of 50%, significantly exceeding the predefined threshold (91). In drug development, organoids, combined with AI and organoid-on-a-chip platforms, can accelerate anti-cancer drug screening and nanomedicine applications, enhancing treatment prediction accuracy (134, 150). Current evidence supports their potential as surrogate endpoints in clinical trials, but further validation through large-scale prospective studies is needed (137, 149, 151).

### 7.3 Inevitability of interdisciplinary integration

The future development of CRC organoid technology depends on deep interdisciplinary convergence. Engineering and materials science contribute by developing Matrigel alternatives (e.g., dSISML) and automated culture platforms (e.g., microfluidic chips and bioprinting) that improve the standardization, throughput, and physiological relevance of organoid production (16, 64, 79). Integration of immunology and microenvironment research is also crucial. Co-culture systems incorporating CAFs, immune cells (e.g., macrophages), and other components enable more realistic simulation of TME complexity and its impact on treatment response (13, 15, 36). Future work should further integrate AI and computational biology, pushing cancer research toward more intelligent, data-driven approaches. Machine learning is used to analyze large organoid phenotypic datasets (e.g., high-content imaging and single-cell transcriptomics) and integrate multi-omics information (transcriptomics, ATAC-Seq) to predict drug responses, decipher resistance mechanisms, build digital twin models, and identify novel biomarkers and therapeutic targets (6, 64, 78, 152). This interdisciplinary integration can address current technical bottlenecks (e.g., insufficient vascularization, limitations in immune microenvironment simulation, and AI model explainability). Ultimately, it aims to build a closed-loop, intelligent organoid platform: from patient sample input and automated culture to AI-driven dynamic monitoring, multi-omics analysis, and digital twin simulation of drug responses, culminating in individualized treatment plans.

**Figure 4** shows a schematic of the closed-loop intelligent organoid platform for colorectal cancer precision medicine.

**Figure 4:**
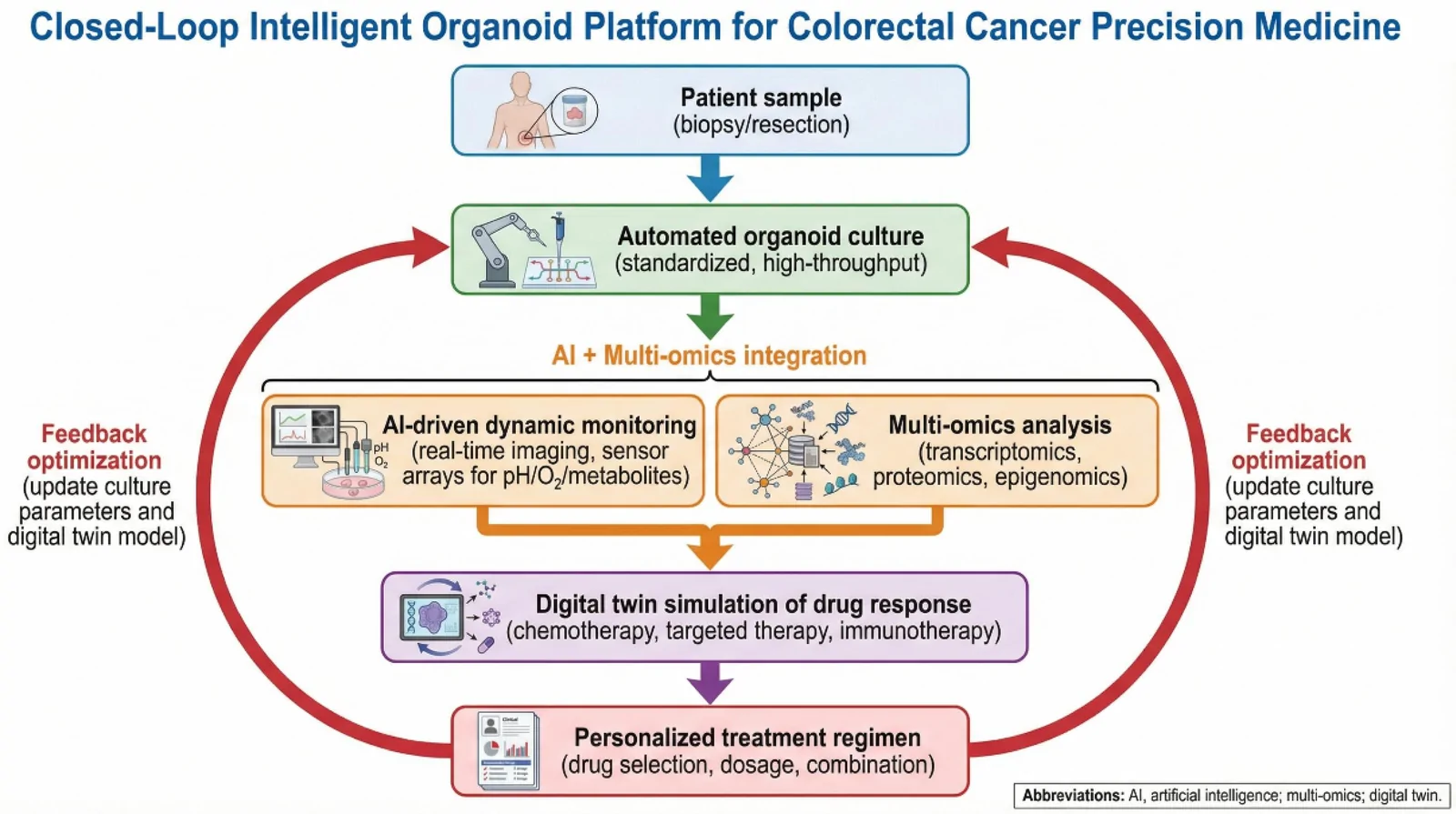
Schematic of the intelligent organoid platform with a closed loop for colorectal cancer precision medicine. The platform integrates automated culture, AI-driven dynamic monitoring, multi-omics analysis, digital twin simulation, and feedback optimization. Patient samples (biopsy or surgical resection) are first processed for automated organoid culture under standardized, high-throughput conditions. AI-based dynamic monitoring (real-time imaging, sensor arrays for pH/O_2_/metabolites) and multi-omics analysis (transcriptomics, proteomics, epigenomics) are performed in parallel. The integrated data then feed into a digital twin model that simulates drug responses to chemotherapy, targeted therapy, and immunotherapy. A personalized treatment regimen (drug selection, dosage, combination) is then generated. A feedback loop from the output back to the culture step enables iterative optimization of both culture parameters and the digital twin model. AI, artificial intelligence.
